# Ablação por Cateter sem Uso de Raios X para Tratamento de Fibrilação Atrial e Arritmias Atriais

**DOI:** 10.36660/abc.20200451

**Published:** 2020-06-29

**Authors:** Leandro Ioschpe Zimerman

**Affiliations:** Hospital de Clínicas de Porto Alegre Porto AlegreRS Brasil Hospital de Clínicas de Porto Alegre – Cardiologia, Porto Alegre, RS – Brasil

**Keywords:** Ablação por Radiofrequência/métodos, Ondas de Radio/efeitos adversos, Equipamento de Proteção Individual, Fluoroscopia, Fistula Arteriovenosa, Eficácia, Segurança

A ablação por radiofrequência é um método consagrado e cada vez mais usado no tratamento das taquiarritmias. Tradicionalmente, é feita por meio da colocação de cateteres intracavitários guiados por fluoroscopia. Ao longo dos anos, no entanto, uma série de problemas relacionados com a exposição à radiação tornou-se mais evidente, tais como catarata, mutações genéticas e câncer.^1^ Não por acaso, a quantidade de tumores no hemisfério cerebral esquerdo, que recebe maior quantidade de radiação, é maior do que no hemisfério direito, em intervencionistas. É importante lembrar que o risco de câncer é linear com a exposição, sem um limiar definido, e que existe efeito cumulativo. Em procedimentos mais longos, lesões graves cutâneas podem, inclusive, se desenvolver nos pacientes. Para reduzir esses riscos aos pacientes e à equipe médica, várias medidas foram tomadas: aparelhos e métodos de fluoroscopia com menor radiação e equipamentos de proteção individual, tais como avental, proteção de tireoide, óculos, touca e até mesmo luvas chumbadas.^2^ A proteção aumentou, mas às custas de problemas ortopédicos pelo peso que se carregava, em tantos procedimentos, por tanto tempo.^3^ Novas soluções foram criadas, como aventais chumbados suspensos. Contudo, junto a esse aumento da proteção individual, foi ganhando força a ideia também de, efetivamente, se realizar o procedimento com a menor quantidade de radiação possível. Para isso, o desenvolvimento de sistemas de mapeamento tridimensional foi o impulso que se precisava. Isso, associado ao uso de cateteres de ablação por força de contato, tornou possível realizar procedimentos, mesmo complexos, manipulando cateteres e aplicando energia com eficácia e segurança, sem necessitar de visualização por fluoroscopia. Em procedimentos menos complexos, especialmente do lado direito do coração, descreviam-se ablações sem fluoroscopia.^4 , 5^ Em gestantes, passou a ser uma solução factível. Mesmo para os procedimentos de maior complexidade, passou-se a preconizar a fluoroscopia “quase zero”. “Quase zero” porque ainda era necessário usar a fluoroscopia em alguns momentos, como a punção transeptal, por exemplo.

Ao mesmo tempo, o ultrassom passou a ser cada vez mais usado em procedimentos cardiológicos invasivos, e mais especificamente em eletrofisiologia. Ecografia vascular é usada para auxílio nas punções vasculares e redução de fístulas arteriovenosas (AV) e pseudoaneurismas. Ecocardiograma transesofágico é útil para excluir trombos em apêndice atrial e auxiliar punção transeptal. Ainda mais útil é o ecocardiograma intracardíaco, que auxilia punção transeptal, permite visualizar óstios de veias pulmonares, descarta derrame pericárdico, visualiza recessos durante ablação do istmo cavotricuspídeo e confirma contato adequado do cateter.

A ideia de que o ultrassom poderia ser usado para substituir o que ainda era feito com fluoroscopia foi descrita há mais de 10 anos, e lentamente vem ganhando espaço na literatura.^6^ No Brasil, o Dr. Eduardo Saad foi um pioneiro no uso do ecocardiograma intracardíaco em ablações complexas, e agora o seu grupo publica a primeira série de casos realizados no Brasil e América Latina com uso zero de fluoroscopia, e sem a necessidade sequer de vestir o avental de chumbo.^7^ Foram 95 pacientes que realizaram o procedimento usando apenas ecocardiograma intracardíaco e mapeamento tridimensional, sendo 69 submetidos à ablação de fibrilação atrial, e incluindo 9 pacientes com marca-passo definitivo. Os procedimentos transcorreram com sucesso e sem complicações maiores. Mesmo as punções transeptais mais difíceis foram realizadas sem o uso de fluoroscopia. “Não foi utilizada fluoroscopia de backup, e nenhum vestuário de chumbo foi necessário“, dizem os autores.

Resultados similares, com sucesso elevado e poucas complicações, têm sido descritos por outros grupos.^8 , 9^ Além disso, trabalhos comparativos têm mostrado que o tempo de aplicação de energia não aumenta, e o sucesso de médio prazo (1 ano) é mantido.^10^ A maior parte dos dados se refere a taquiarritmias supraventriculares, mas também apresenta bons resultados em ablações de extrassístoles e taquicardias ventriculares.^11^

Ao mesmo tempo que o conceito de que é possível e desejável realizar os procedimentos sem usar a fluoroscopia é aceito e se torna a norma, passam a ser buscadas outras técnicas, além da associação do mapeamento tridimensional com o ecocardiograma intracardíaco. Estudos recentes descrevem a realização de punção transeptal e ablação de fibrilação atrial sem o uso do ecocardiograma intracardíaco, mas usando sa agulhas de punção transeptal como “cateter bipolar”,^12^ ou identificando a fossa *ovalis* somente com o sistema de mapeamento 3-D.^13^

Não parece haver dúvidas de que o futuro aponta para ablações sem o uso de fluoroscopia, até mesmo porque questões éticas e jurídicas deverão impulsionar nessa direção. No entanto, quais são, atualmente, os obstáculos que dificultam o uso regular da técnica? Em primeiro lugar, é preciso lembrar situações nas quais ainda não se testou a técnica, e que parece de mais difícil aplicação, como na ablação epicárdica ou em arritmias relacionadas com cardiopatia congênita complexa. Em segundo lugar, o custo. Não são todos os seguros que cobrem o uso do ecocardiograma intracardíaco, e a maior parte dos pacientes não tem condições de arcar com esse custo. Em terceiro lugar, a inércia. A maior parte dos eletrofisiologistas está acostumada com as técnicas tradicionais (que obtêm bons resultados) e não está disposta a passar por uma nova curva de aprendizado. Para procedimentos como ablação de fibrilação atrial, não parece que esses obstáculos se sustentem frente aos benefícios da técnica. Creio que fica a dúvida com relação aos procedimentos de baixa complexidade, como a ablação de taquicardia supraventricular, que hoje são feitos com alto sucesso, raríssimas complicações e doses muito baixas de radiação. Se o ganho que se obtém com o mapeamento tridimensional e uso do ecocardiograma intracardíaco compensa o custo e a necessidade de se colocar uma bainha mais calibrosa para a sonda do ecocardiograma, ainda deverá ser mais bem-definido.

A ablação sem fluoroscopia é um grande avanço, e já está pronta para ser implementada em grande escala. Contudo, como ocorre com todo grande avanço na ciência, já aguarda o próximo passo na evolução. Os procedimentos em geral estão sendo realizados progressivamente de formas menos invasivas. Cirurgias abertas são substituídas por procedimentos por cateteres e videolaparoscopia. Na área das arritmias cardíacas, ablações já estão sendo feitas sem a necessidade da colocação de cateteres, mas usando mapeamento por sistemas de eletrodos externos, e ablação por estereotaxia, com radiação por feixe externo (como a radioterapia). Inicialmente desenvolvida e descrita no tratamento das taquicardias ventriculares,^14^ a técnica agora engatinha também para ser usada na ablação de fibrilação atrial.^15^

A ablação é terapêutica indispensável e vai se manter como tratamento usual para taquiarritmias. A fluoroscopia, por sua vez, é danosa e vai ser eliminada por procedimentos eletrofisiológicos. O assunto é premente, e o interesse é mundial. É hora de aposentar o avental de chumbo. Aqui, ao contrário do que se costuma popularmente dizer, quanto menos, melhor. E se for zero, melhor ainda.
