# Errata

**DOI:** 10.36660/abc.20210747

**Published:** 2021-09-01

**Edição de Junho de 2020, vol. 114 (6), págs. 1027-1028**

No Minieditorial “Ablação por Cateter sem Uso de Raios X para Tratamento de Fibrilação Atrial e Arritmias Atriais”, com número de DOI: https://doi.org/10.36660/abc.20200159, publicado no periódico Arquivos Brasileiros de Cardiologia, 114(6):1027-1028, na página 1027, alterar o DOI para: https://doi.org/10.36660/abc.20200451.

