# Supplementary material for: Ablação por Cateter sem Uso de Raios X para Tratamento de Fibrilação Atrial e Arritmias Atriais
Source: Arq Bras Cardiol. 2020 Jun 29;114(6):1027–8. [Article in Portuguese] doi: 10.36660/abc.20200451 (PMC8416118; doi:10.36660/abc.20200451)
Supplement: Translation

# Translation: Catheter Ablation Without Use of X-rays to Treat Atrial Fibrillation and Atrial Arrhythmia

**Authors:** Leandro Ioschpe Zimerman

**Affiliations:** Brazil Hospital de Clínicas de Porto Alegre – Cardiologia, Porto Alegre, RS – Brazil

**Keywords:** Radiofrequency Ablation/methods, Radio Waves/adverse effects, Personal Protective Equipment, Fluoroscopy, Arteriovenous Fistula, Efficacy, Safety

Radiofrequency ablation is a well-established method and is increasingly used in the treatment of tachyarrhythmia. Traditionally, it is done by placing fluoroscopy-guided intracavitary catheters. Over the years, however, a number of problems related to exposure to radiation have become more evident, such as cataracts, genetic mutations, cancer.^1^ It is not by chance that the number of tumors in the left cerebral hemisphere, which receives the greatest amount of radiation, is greater than in the right hemisphere, in interventionists. It is important to remember that the risk of cancer is linear with exposure, without a defined threshold, and that there is a cumulative effect. In longer procedures, even severe skin lesions can develop in patients. To reduce these risks to patients and the medical staff, several actions were taken: fluoroscopic devices and methods with less radiation, personal protective equipment, such as an apron, thyroid protection, goggles, cap and even leaded gloves.^2^ Protection increased, but at the expense of orthopedic problems due to the weight that was carried, in so many procedures, for so long.^3^ New solutions were created, such as suspended lead aprons. But along with this increase in personal protection, the idea of effectively performing the procedure with the least amount of radiation possible gained momentum. For this, the development of three-dimensional mapping systems was the driving force required. This, associated with the use of ablation catheters by contact, made it possible to perform procedures, including complex ones, manipulating catheters and applying energy efficiently and safely, without requiring fluoroscopic imaging. In less complex procedures, especially on the right side of the heart, ablations without fluoroscopy were described.^4 , 5^ In pregnant women, it has become a feasible solution. Even for the more complex procedures, “near-zero” fluoroscopy was recommended. “Near-zero” because it was still necessary to use fluoroscopy at times, such as transseptal puncture, for example.

At the same time, ultrasound has been increasingly used in invasive cardiac procedures and, more specifically, in electrophysiology. Vascular ultrasound is used to support vascular punctures and to reduce AV fistulas and pseudoaneurysms. Transesophageal echo is useful to rule out atrial appendage thrombus and to assist transseptal puncture. Even more useful is intracardiac echo, which assists transseptal puncture, shows pulmonary vein ostia, rules out pericardial effusion, shows recesses during cavotricuspid isthmus ablation and confirms adequate catheter contact.

The idea that ultrasound could be used to replace what was still done with fluoroscopy was described more than 10 years ago, and it is slowly gaining space in the literature.^6^ In Brazil, Dr. Eduardo Saad was the first to use intracardiac echocardiography in complex ablation procedures, and now his group publishes the first series of cases performed in Brazil and Latin America with zero use of fluoroscopy, which does not even require the lead apron.^7^ Ninety-five patients underwent the procedure using only intracardiac echocardiography and three-dimensional mapping, 69 of whom underwent atrial fibrillation ablation, including 9 patients with permanent pacemaker. The procedures were carried out successfully and without major complications. Even the most difficult transseptal punctures were performed without fluoroscopy. “Backup fluoroscopy was not used, and no lead clothing was needed,” the authors say.

Similar results with high success and few complications have been described by other groups.^8 , 9^ Moreover, comparative studies have shown that the energy application time does not increase and medium-term success (1 year) is maintained.^10^ Most of the data refer to supraventricular tachyarrhythmias, but they also have good results for ablation of extrasystole and ventricular tachycardia.^11^

While the concept that it is possible and desirable to do the procedures without fluoroscopy is accepted and becomes the norm, other techniques are sought, in addition to the association of three-dimensional mapping with intracardiac echocardiography. Recent studies describe the performance of transseptal puncture and atrial fibrillation ablation without intracardiac echocardiography, but using transseptal puncture needles such as a “bipolar catheter”,^12^ or identifying the oval fossa with 3-D mapping only.^13^

There seems to be no doubt that the future points to ablation without fluoroscopy. Even because ethical and legal issues should push in this direction. But what are the obstacles to the regular use of this technique today? Firstly, it is necessary to have in mind the situations in which the technique has not yet been tested and where it seems to be more difficult to apply, such as in epicardial ablation or in arrhythmias related to complex congenital heart diseases. Secondly, the cost. Not every insurance covers the use of intracardiac echocardiography, and most patients are unable to afford this cost. Thirdly, inertia. Most electrophysiologists are used to traditional techniques, get good results, and are not willing to go through a new learning curve. For procedures such as atrial fibrillation ablation, these obstacles do not appear to outweigh the benefits of the technique. I believe that the doubt remains regarding low-complexity procedures, such as ablation of supraventricular tachycardia, which has been successfully performed, with very rare complications and very low doses of radiation. If the gain obtained with three-dimensional mapping and use of intracardiac echocardiography outweighs the cost and the need to place a more calibrated sheath for the echocardiogram probe, it is yet to be better defined.

Ablation without fluoroscopy is a major progress and is now ready to be implemented on a large scale. But, as with every major progress in science, it is already waiting for the next step in evolution. The procedures in general are being performed progressively in less invasive ways. Open surgeries are replaced by catheter and laparoscopic procedures. As for cardiac arrhythmias, ablations have been performed without the need for catheter placement, but using mapping by external electrode systems, and stereotaxis ablation, with external beam radiation (such as radiotherapy). Initially developed and described in the treatment of ventricular tachycardias,^14^ the technique is now also potentially to be used in ablation of atrial fibrillation.^15^

Ablation is an indispensable therapy and will remain as such as the usual treatment for tachyarrhythmia. Fluoroscopy is harmful and will be eliminated by electrophysiological procedures. This is a pressing issue and concerns the whole world. It is time to dispense with the lead apron. Here, as opposed to what is popularly said, the less the better. And if it’s zero, that’s even better.
