# Supplementary material for: Errata
Source: Arq Bras Cardiol. 2021 Sep 1;119(6):1009. [Article in Portuguese] doi: 10.36660/abc.20210747 (PMC9814795; doi:10.36660/abc.20210747)
Supplement: Translation

# Translation: Erratum

**June 2021 Issue, vol. 114 (6), pages 1027-1028**

In Short Editorial “Catheter Ablation Without Use of X-rays to Treat Atrial Fibrillation and Atrial Arrhythmia” with DOI: https://doi.org/10.36660/abc.20200159, published in the Journal Arquivos Brasileiros de Cardiologia, 114(6):1027-1028, on page 1027, change DOI to: https://doi.org/10.36660/abc.20200451.
